# An Unusual Case of Asystole following Penetrating Neck Trauma and Anoxic Brain Injury

**DOI:** 10.1155/2011/579805

**Published:** 2011-07-07

**Authors:** Matthew Nayor, Alissa J. Berliner, Grant V. Chow, David D. Spragg

**Affiliations:** ^1^Department of Medicine, Johns Hopkins Medical Institutions, Johns Hopkins University School of Medicine, Baltimore, MD 21287, USA; ^2^CCBC Essex and College of Health Professions, Towson University, Towson, MD 21252-0001, USA; ^3^Division of Cardiology, Johns Hopkins Medical Institutions, Johns Hopkins University School of Medicine, Baltimore, MD 21287, USA

## Abstract

Bradycardia and transient asystole are well-described sequelae of a myriad of neurologic insults, ranging from focal to generalized injuries. Increased vagal tone also predisposes many individuals, particularly adolescents, to transient neurally mediated bradyarrhythmia. However, prolonged periods of sinus arrest without junctional or ventricular escape are quite rare, even after significant neurologic injury. We describe the case of a 17-year-old man who presented with anoxic brain injury secondary to hemorrhagic shock from a stab wound to the neck. His recovery was complicated by prolonged periods of sinus arrest and asystole, lasting over 60 seconds per episode. This case illustrates that sustained asystolic episodes may occur following significant neurologic injury, and may continue to recur even months after an initial insult. Pacemaker implantation for such patients should be strongly considered.

## 1. Introduction

Neurally mediated bradycardia and brief periods of asystole are commonly observed in the setting of head injury, and may also be induced clinically by performance of vagal maneuvers or deliberate positional change. In general, these episodes are classified as “benign,” due to their transient and self-limited nature. Contrary to common experience, however, we report the case of a 17 year old man whose recovery following anoxic brain injury was complicated by episodes of unprovoked, prolonged periods of asystole. 

## 2. Case Report

A 17 year old man was found unresponsive, pulseless, and bleeding profusely from a stab wound to the neck. Emergency medical services were called, and cardiopulmonary resuscitation (CPR) was initiated for pulseless electrical activity (PEA) arrest. A palpable pulse was restored after one minute of chest compressions, epinephrine, atropine, and intravenous saline administration. The patient was subsequently intubated for airway protection.

On arrival to the emergency department, the patient was unconscious, with a heart rate of 85 bpm and blood pressure of 133/87 mm Hg. He was taken to the operating room for emergent surgical exploration, which revealed a 2 cm left-sided neck wound, penetrating to the deep fascia of the sternocleidomastoid muscle, and with no apparent damage to the airway or major blood vessels. 

Emergent computed tomography (CT) of the head revealed a subarachnoid hemorrhage in the posterior fossa, with a small amount of bleeding in the right foramen of Luschka and the posterior aspect of the fourth ventricle. CT angiography of the head and neck revealed patent vasculature, without evidence of mass effect or carotid body distortion. There was no local hematoma formation, nor was there significant regional swelling. Further evaluation of the brain by magnetic resonance imaging (MRI) confirmed acute anoxic brain injury, with ischemic changes noted in bilateral middle cerebral artery and anterior cerebral artery territories. An electroencephalogram (EEG) demonstrated generalized epileptiform discharges, and the patient was diagnosed with postanoxic status epilepticus.

**Figure 1:**
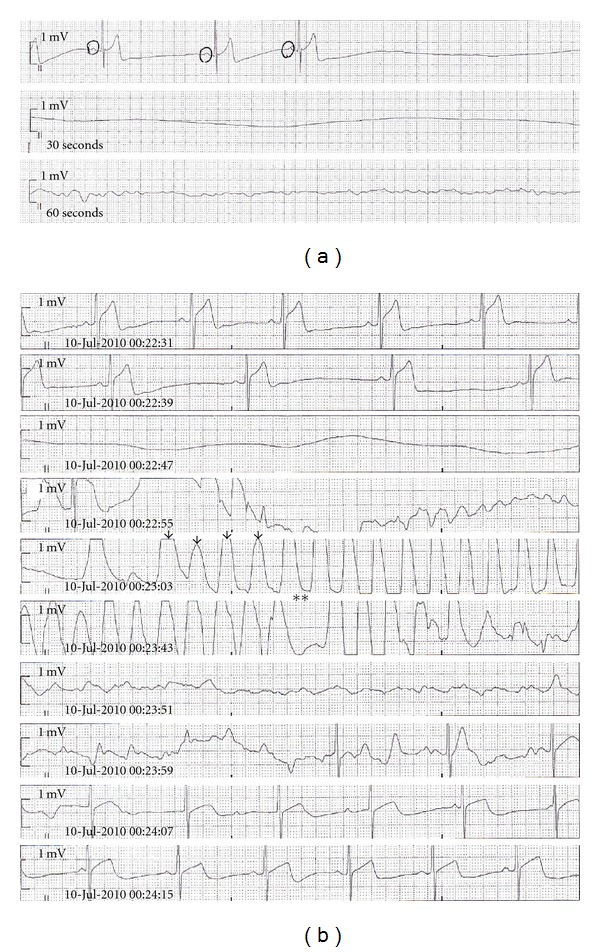
(a) Sinus bradycardia progressing to sinus arrest and asystole. (b) Recurrent sinus arrest, asystole, chest compression artifact (arrows), and eventual restoration of sinus rhythm. Asterisks (**) represent approximately 30 seconds of elapsed time.

The patient was admitted to the Neurologic Critical Care Unit, where he was stabilized and initiated on a hypothermia protocol for anoxic brain injury. This protocol was halted after two episodes of marked bradycardia progressing to asystole, each resolving after one minute of chest compressions before epinephrine or atropine could be given. Review of telemetry data revealed sinus arrest without atrial, junctional, or ventricular escape (Figure 1(a)). After resuscitation, serial electrocardiograms (ECGs) demonstrated normal sinus rhythm.

The patient had a prolonged hospital course, during which his neurologic status remained poor. Seizures were controlled with phenytoin, modafinil, and levetiracetam. On hospital day number 36, there was recurrence of sinus bradycardia that progressed to asystolic arrest, which resolved after several seconds. Modafinil was discontinued and phenytoin was changed to valproic acid, in the event that this episode represented a toxic side effect. Phenytoin levels at the time were checked, and were low (<10 mcg/mL). 

On hospital day number 46, the patient suffered sequential periods of asystolic arrest, each of which required chest compressions, and which resolved spontaneously after 60 seconds (Figure 1(b)). The decision was made to place a single-lead ventricular pacemaker for definitive treatment, programmed to VVI with a base rate of 50 beats per minute without hysteresis or rate drop response. 

The patient slowly improved from a neurologic standpoint, and was discharged to a chronic care facility. Subsequent device interrogation six months after implantation revealed numerous episodes of bradycardia with appropriate demand pacing at 50 beats per minute. There was no evidence of associated hypotension with these episodes. 

## 3. Discussion

We report a case of recurrent, episodic sinus bradycardia and asystolic arrest in a patient with traumatic neck injury and anoxic encephalopathy, occurring up to six months after the initial insult. Bradycardia and asystole are well-described consequences of numerous neurologic insults, ranging from generalized to focal injuries, as well as in association with certain medications. Further, increased vagal tone is a common cause of neurally-mediated, transient bradyarrhythmia. 

Of the mechanisms related to generalized neurologic insults, the Cushing reflex is perhaps the most commonly known. It is characterized by hypertension, bradycardia, and apnea in a patient with increased intracranial pressure and is thought to be a protective regulatory mechanism to maintain adequate cerebral perfusion [1]. Seizures may also be associated with autonomic dysfunction and arrhythmia. While ictal tachycardia is more common, bradycardia and asystole have been described as a rare complication of temporal lobe seizures [2]. 

Certain focal neurologic injuries have also been implicated in causing bradyarrhythmias. Stimulation of sublobule IX-b of the cerebellar vermis has been shown in animal models to cause bradycardia, hypotension, and decreased phrenic nerve stimulation via decreased sympathetic tone [3]. Stimulation of the trigeminal nerve (usually during a surgical procedure) has been shown to activate the trigeminocardiac reflex, causing bradycardia, hypotension, apnea, and gastric hypermotility [1]. Bradycardia and asystole as sequelae of cervical spinal injuries have also been described. These result from interruption of the sympathetic innervation of the heart, which exits the spinal cord at T1-T4 [4]. 

Two of the medications that our patient was prescribed, phenytoin and modafinil, have been associated with bradycardia. Intravenous phenytoin is known to cause severe bradycardia and hypotension with rapid infusion [5]. Oral formulations are less likely to cause significant bradycardia, and are infrequently associated with any adverse hemodynamic effects. However, there are case reports of oral phenytoin overdose causing junctional rhythms and bradycardia [6] in association with markedly elevated serum phenytoin levels. Similarly, modafinil has been rarely associated with bradycardia. In one study, 1 out of 175 patients taking modafinil for up to one year developed mild bradycardia and syncope with no other obvious precipitants [7]. 

Increases in vagal tone and activation of the Bezold-Jarisch baroreflex are perhaps the most common neurally-mediated causes of bradycardia, slowed intracardiac conduction, and syncope [8]. Bradycardia as a result of endotracheal suctioning has been well described and is likely secondary to stimulation of vagal afferent fibers as well as an effect of hypoxemia [9]. A rare, but extreme form of malignant vagotonia is termed “Jordan syndrome,” characterized by interrupted afferent input from the carotid sinus to the nucleus tractus solitarii while efferent parasympathetic output remains intact. Severe bradycardia, asystole, and hypotension may result [10, 11]. Typically, though, these patients have periods of unchecked sympathetic output as well (with attendant hypertension and tachycardia); this phenomenon was not seen in our patient. Finally, sinus arrest has also been described during rapid eye movement (REM) sleep in otherwise healthy young adults, and is thought to be secondary to increased vagal tone [12]. 

Our patient was initially admitted following penetrating neck injury and PEA arrest, complicated by status epilepticus and myoclonus. Although his episodes of bradycardia and sinus arrest began in the setting of hypothermia and generalized neurologic injury, they also recurred after rewarming, and were independent of any seizure activity. After extensive neurologic imaging, no focal lesions known to correlate with bradyarrhythmias were found. Upon review of administered medications, our patient was receiving the oral suspension of phenytoin via gastrostomy tube, but levels did not approach a toxic range. Further, asystolic episodes continued to recur despite discontinuation of both phenytoin and modafinil, and were also noted months after discharge. 

We suspect, therefore, that our patient represents an unusual case of isolated malignant vagotonia, likely stemming from anoxic cerebral injury. Long-term pacing support for such patients may be necessary.
